# Effective Size Reduction of the Metallic Waveguide Bandpass Filter with Metamaterial Resonators and Its 3D-Printed Version

**DOI:** 10.3390/s23031173

**Published:** 2023-01-19

**Authors:** Junghyun Cho, Yejune Seo, Jihaeng Cho, Kyoung Youl Park, Joongki Park, Hosub Lee, Sungtek Kahng

**Affiliations:** 1 Department of Information & Telecommunication Engineering, Incheon National University, Incheon 22012, Republic of Korea; 2 Agency for Defense Development, Daejeon 34186, Republic of Korea; 3 LIGNEX1, Seongnam 13488, Republic of Korea

**Keywords:** waveguide, bandpass filter, metamaterial, equivalent circuit, metamaterial structure, zeroth-order resonance, 3D printing

## Abstract

In this paper, a novel method is proposed to effectively reduce the size of a waveguide bandpass filter (BPF). Because the metallic cavities make the conventional waveguide end up with a large geometry, especially for high-order BPFs, very compact waveguide-type resonators having metamaterial zeroth-order resonance (WG ZOR) are designed on the cross section of the waveguide and substituted for the cavities. While the cavities are half-wavelength resonators, the WG ZOR is shorter than one-eighth of a wavelength. A substantial reduction in the size and weight of the waveguide filter is observed as the resonators are cascaded in series through coupling elements in the X-band that is much longer than that in K- or Ka-bands. The proposed metamaterial filter is realized as a 3D-printed structure to be lighter and thus more suitable for low earth orbit (LEO) satellites. An X-band of 7.25–7.75 GHz is chosen to verify the method as the passband with an attenuation of 40 dB at 7.00 GHz and 8.00 GHz as the roll-off in the stopband. The BPF is manufactured in two ways, namely the CNC-milling technique and metal coating–added 3D printing. The design is carried out with a geometrical parameter of not 10^−2^ mm but rather 10^-1^ mm, which is good for manufacturers but challenging for component designers. The measurement of the manufactured metal waveguide filters reveals that the passband has about ≤1 dB and ≤−15 dB as the insertion loss and the reflection coefficient, respectively, and the stopband has an attenuation of ≤−40 dB, which are in good agreement with the results of the circuit and the simulation. The proposed filter has a length of 14 cm as the eighth-order BPF, but the conventional waveguide is 20 cm as the seventh-order BPF for the same area of the cross section.

## 1. Introduction

Artificial satellites are watching the earth for global-scale wireless networking and scientific data gathering. With telecommunication and weather surveillance, satellites are indispensable to the surveillance and reconnaissance of target regions. Nowadays, LEO satellites are produced for commercial services and deployed in orbit 800 km to 1000 km above the earth’s surface, which comes with a short cycle of production and which forms a space-borne network of an overwhelming number of satellites, called a constellation [1,2]. Transponders are essential to satellites of any kind and any missions for ground-to-space and satellite-to-satellite wireless links.

The transponder is a wireless communication system that receives RF signals and transmits them, whether it is geostationary or on a LEO satellite. Its configuration is represented by the system block diagram comprising plenty of circuits and components, from the receiver to the transmitter. The chain of the signal flow meets the components of signal amplification, channel selection, channel splitting and combining, switching, demodulation and modulation, etc. For successful tasking, system integration (SI) is important, but the functions of the individual components are more prioritized. Active components connected to control blocks have an increased degree of freedom in meeting the requirements even after SI. As for passive components such as and input and output multiplexers (MUXes) for channel splitting and combining, and filters for channel selection, functions are not controllable after fabrication and assembly. Great care should be taken of their design and fabrication. While active components such as MMIC amplifiers do not weigh much, the passive components of waveguide (WG) filters and WG-MUXes tend to be heavy. Along with the feed horn and reflector antenna, WG passive elements are of great concern in terms of size control and weight control in the building of a satellite transponder. This becomes a critical matter to the cost required in the making and maintaining of LEO satellites and microsatellites.

In satellite communication, high-frequency signals must be so strong that they can travel hundreds to thousands of kilometers when emanated from the antenna. The filters, like other passive components in the feed assembly of the antenna, are made out of metallic waveguides to ensure high Q-factors and endure high power and the heat [3,4,5,6,7]. This is why the structures are bulky and heavy. Reducing their size is a crucial factor to saving on costs in developing and launching satellites. Choocadee and Akatimagool showed second- and third-order rectangular WG BPFs [3]. Typical-size cavities are combined in series through irises for channel selectivity. San-Blas and Boria took action to reduce the size of the WG filter by integrating coaxial lines with rectangular cavities [4]. The length of their third-order filter is the same as that of the conventional third-order BPFs. Valencia and Cogollos put in SIR parts with cavity resonators coupled through a mixture of E-plane and H-plane steps [5]. Their WG BPF is longer than four wavelengths. Teberio and Percaz meandered the straight topology of a BPF to reduce the area that the filter occupies [6]. The added waveguide to the mitered E-plane bend sections couple with half-wavelength cavities, and the total length has almost no change. Sullca and Guglielmi changed a straight geometry to a staircase by opening the two spots on the broad wall of each cavity for coupling [7]. The trace in the longitudinal axis is decreased, but the total length is similar to that in [5]. overran overview of the latest rectangular waveguide bandpass filters, including the reports above, as they follow the design technique of half-wavelength cavities, shows that effective size reduction is nowhere to be found. One might suggest applying metamaterial filter designs in microstrip and CPW lines, such as those in [8,9], to waveguide components, but this turns out to be not possible, because of differences in geometries and modes. Another idea might be to set up a metamaterial resonator, which is much shorter than the typical cavity and geometrically appropriate for the waveguide.

This paper proposes the novel design method of realizing the compact WG BPF of a high order for a satellite communication system by devising and using a waveguide metamaterial resonator. First, the zeroth-order resonator is formed on the cross section of the standard waveguide, compliant with the CRLH circuit model. The geometrical parameters are of 10^−1^ mm to avoid the problems of fabrication tolerance, whose lower precision makes this design much tougher than others, using 10^-2^ mm, with more expensive facilities. Second, the ZOR as a building block for the equivalent network of the eighth-order BPF is substituted for conventional cavities. Third, the resonators are cascaded through short waveguide sections as coupling elements to generate the passband. Additionally, the stopbands have high attenuation. Fourth, the WG BPF of ZORs and coupling elements is physically prototyped by the CNC-milling technique. Fifth, the metallic WG filter is transformed into a 3D-printed version to lower the weight of the structure, potentially proper for fast and mass production. According to [10,11,12], this is the first 3D-printed metamaterial waveguide bandpass filter that has been made by the PC PEKK-based additive process. Sixth, its performance is tested. The suggested method is evaluated by circuit modeling, full-wave EM simulation and measurement. Good agreement between the theoretical results and the experimental results is unveiled from the procedural steps. As noted in the required specifications on the WG BPF, the passband has about ≤1 dB and ≤−15 dB as the insertion loss and the reflection coefficient, respectively; the stopband has 40 dB of noise suppression, whereas its 3D-printed version has a little degraded insertion loss and return loss owing to material loss and surface roughness. Regarding size reduction, the proposed PBFs of metallic and 3D-printed waveguides are 14 cm for the eighth-order filtering, while the conventional waveguide filter of the seventh order is of 20 cm in length. This effect will be obvious for much-higher-order filters and multiplexers.

## 2. Circuit Modeling of the BPF and the WG ZOR, and Its Geometry

### 2.1. Required Specifications of the BPF for Satellite Wireless Communication

A bandpass filter is needed in the transponder as the communication system for the satellites with the following assessment items and values.

The mathematical approach discovers that the function of the Chebyshev type with *N*_order_ = 8 is quite close to the specifications in Table 1 for the amplitude of the bandpass filter, as in [13]. It is expressed with the circuit network.

**Figure 1:**
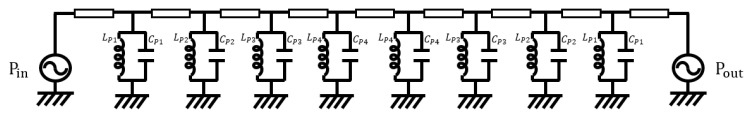
Equivalent circuit network of the bandpass filter.

Figure 1 has eight parallel LC resonators cascaded through transmission-line segments as coupling elements. Equation 1 is the transfer function of the curve fitting the amplitude. The long expression is decomposed into a few terms. The maximum order of the denominator of *t*(*s*) is the same as the number of the resonators. As is performed for filter designs, the transmission-line segments or waveguide sections of nearly a quarter wavelength work as an inductor mentioned in [14] and are put between the resonators for the bandwidth. In the PCB filters and waveguide filters, the LC resonators seen in Figure 1 are changed to the distributed elements, the lengths of which are half wavelength and odd multiple half wavelengths. This is the conventional rule that is most often used, thanks to its simplicity and convenience. If and only if this rule is kept, the bandpass filter will end up with a long structure when a higher-order filtering is required, like the specifications in Table 1. The configuration of Figure 1 with the parallel LC resonators leading to conventional resonators is modified into something with new resonators as follows.

**Figure 2:**
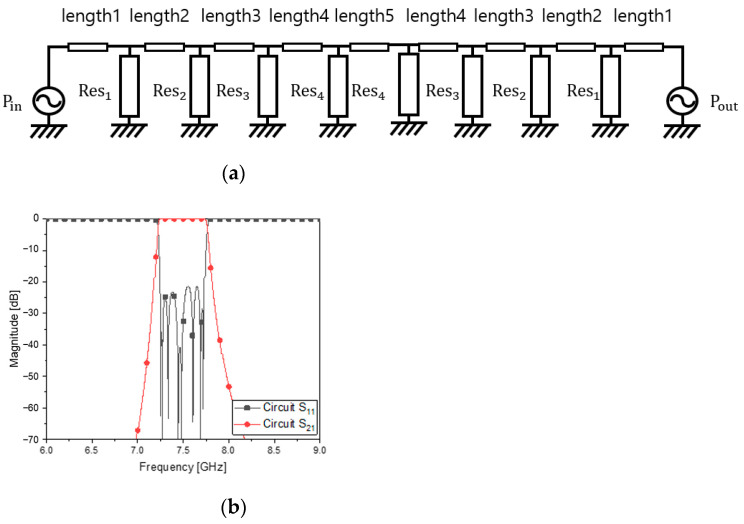
Modified equivalent circuit and its frequency response: (**a**) circuit (**b**) S_11_ and S_21_.

The resonators of Figure 2a will be filled with new ones. By satisfying the requirement, the circuit calculation gives the unknowns in Figure 1 and Figure 2 and the values in Table 2. Figure 2b presents S_11_ as the reflection coefficient and S_21_ as the transmission coefficient of the device. This ideal circuit results in the satisfactory frequency response. The circuit calculation is operated on the basis of the following mathematical procedures.

(1) $$t\left(s\right)=\frac{5.356*10^{8}s-8.871*10^{19}}{s^{2}+1.158*10^{9}s+2.193*10^{21}}+\frac{5.896*10^{8}s+9.092*10^{19}}{s^{2}+1.172*10^{9}s+2.248*10^{21}}\;+\frac{-1.08*10^{9}s+4.22*10^{19}}{s^{2}+8.982*10^{8}s+2.138*10^{21}}+\frac{-1.14*10^{9}s-4.552*10^{19}}{s^{2}+9.329*10^{8}s+2.306*10^{21}}\;+\frac{7.573*10^{8}s-5.793*10^{18}}{s^{2}+5.807*10^{8}s+2.095*10^{21}}+\frac{8.043*10^{8}s-6.506*10^{18}}{s^{2}+6.153*10^{8}s+2.353*10^{21}}\;+\frac{-2.253*10^{8}s-3.449*10^{18}}{s^{2}+2.015*10^{8}s+2.073*10^{21}}+\frac{-2.41*10^{8}s+3.958*10^{18}}{s^{2}+2.159*10^{8}s+2.379*10^{21}}$$

(2) $$\left[ABCD_{Tot.}^{Circ.}\right]=\left[ABCD_{Res.1}^{Circ.}\right]\left[ABCD_{m_{12}}^{Circ.}\right]\left[ABCD_{Res.2}^{Circ.}\right]\cdots \left[ABCD_{m_{78}}^{Circ.}\right]\left[ABCD_{Res.8}^{Circ.}\right]\;\left[ABCD_{Res.1}^{Circ.}\right]=\left[ABCD_{L1}^{Circ.}\right]\left[ABCD_{C1}^{Circ.}\right]=\left[\begin{array}{cc} 1 & 0\\ 1/jwL1 & 1 \end{array}\right]\left[\begin{array}{cc} 1 & 0\\ jwC1 & 1 \end{array}\right]\;\left[ABCD_{m12}^{Circ.}\right]=\left[\begin{array}{cc} cos\beta l & jZ_{0}sin\beta l\\ jY_{0}sin\beta l & cos\beta l \end{array}\right],\;where\;l=length2\;\left[ABCD_{Tot.}^{Circ.}\right]=\left\{\prod_{p=1}^{N-1}\left[ABCD_{Res.p}^{Circ.}\right]\left[ABCD_{m_{p,p+1}}^{Circ.}\right]\right\}\cdot \left[ABCD_{Res.N}^{Circ.}\right]\;\;=\left[\begin{array}{cc} A_{Tot.} & B_{Tot.}\\ C_{Tot.} & D_{Tot.} \end{array}\right]\;S_{11}^{Circ.}=\frac{A_{Tot.}+B_{Tot.}/Z_{0}-C_{Tot.}Z_{0}-D_{Tot.}}{A_{Tot.}+B_{Tot.}/Z_{0}+C_{Tot.}Z_{0}+D_{Tot.}}\;S_{21}^{Circ.}=\frac{2}{A_{Tot.}+B_{Tot.}/Z_{0}+C_{Tot.}Z_{0}+D_{Tot.}}$$

The ABCD-parameter matrix of each block of the circuit is sequentially multiplied from *P_in_* to *P_out_*. S_11_ and S_21_ are functions of the elements of the finalized ABCD matrix.

### 2.2. Devising the Metamaterial Resonators in the Waveguide

The making of a thin resonator within the cross section of the waveguide starts at the equivalent circuit modeling for the zeroth-order resonator. Unlike the previous ZOR filters or metamaterial passive devices, which are formed mainly in the longitudinal direction as the microstrip line or CPW, the novel ZOR is proposed to be formed in the transverse directions on the WG cross section as a novel approach. A CRLH circuit is built by considering the up, down, left and right metallic walls.

**Figure 3:**
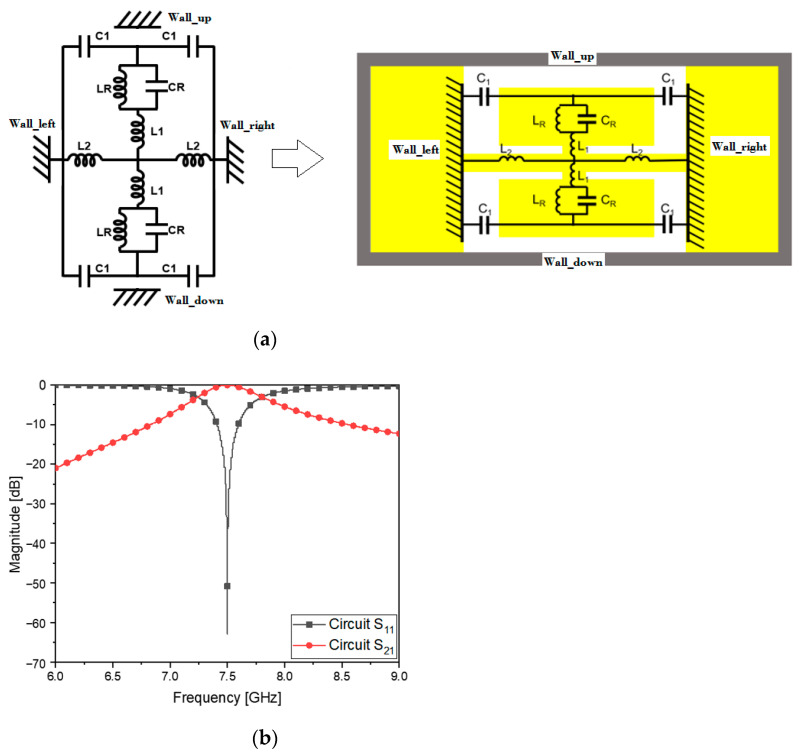

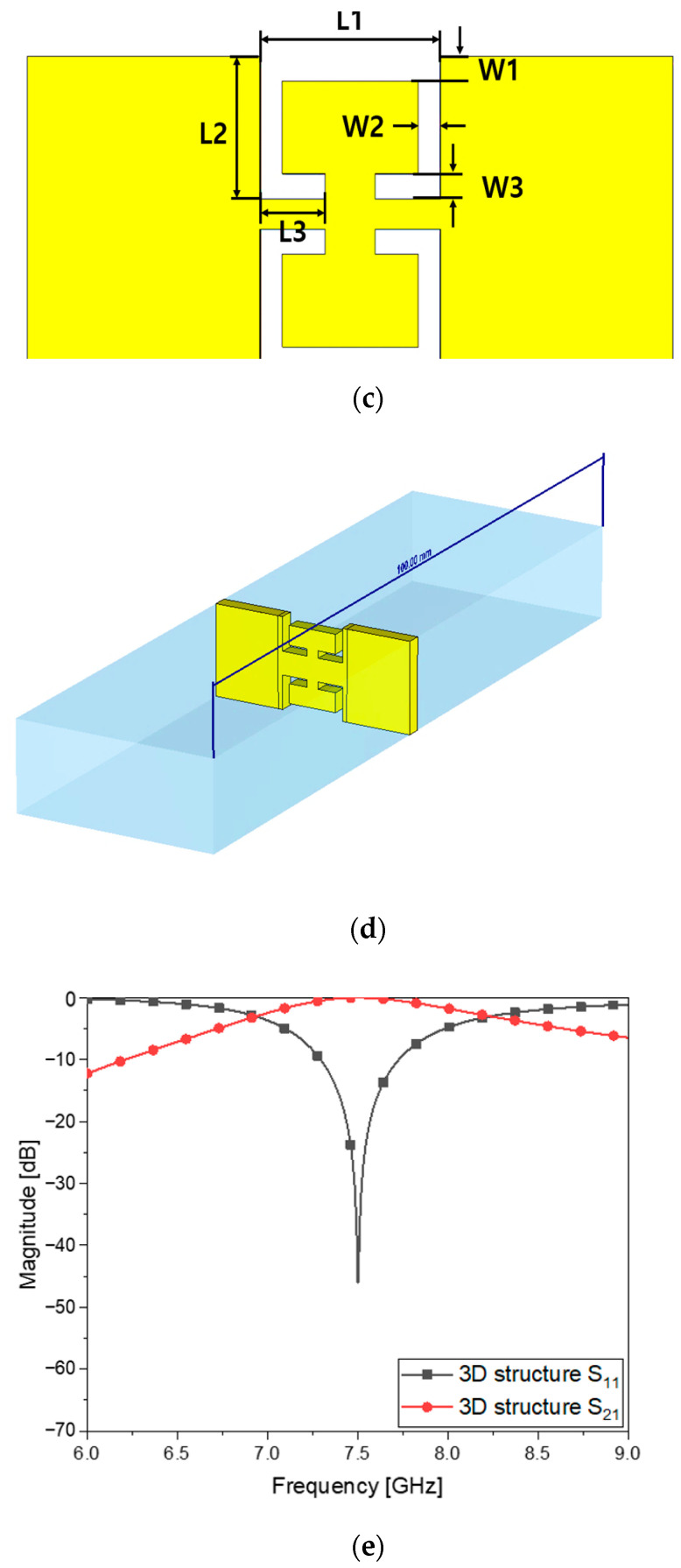
Circuit model of the CRLH resonator and moved into the waveguide. (**a**) E-CRLH circuit of the resonator applied to the waveguide cross section; (**b**) S_11_ and S_21_ of the resonator circuit; (**c**) physical shape of the thin resonator; (**d**) the resonator is longitudinally flat; (**e**) S_11_ and S_21_ of the resonator structure.

In Figure 3a, the E-CRLH circuit is given as a combination of shunt L, shunt C, series L and series C, which go well with four metallic surfaces on the waveguide cross section. C_1_ as series C from *Wall_left* to *Wall_right* and L_2_ as shunt L from *Wall_left* to *Wall_right* are slots and short-circuited with the metal walls, spread in the transvers directions. In the vertical electric field of the TE_10_ mode, a metal plate gets in the way to capture the E-field and is divided into the upper and lower patches modeled as L_R_C_R_ subresonators connected through a strip equivalent to L_1_. Combining the electrical attributes of the elements, for the purpose of the metamaterial resonance at the center frequency, the circuit values are obtained as follows:

By using the values in Table 3, S_21_ and S_11_ in the plot of Figure 3b show the resonance as planned.

As mentioned, the geometrical information of the slots, short-circuiting lines, metal patches and their connecting strip in Figure 3c,d is determined by electromagnetically simulating the structure in a full-wave analysis program, on the basis of C_1_, L_2_, L_R_C_R_ and L_1_ to obtain the same frequency response as Figure 3b. As a result, S_21_ and S_11_ of the flat WG metamaterial resonator of the physical dimensions written in Table 4 can be obtained when Figure 3e agrees with Figure 3b. Using the frequency response and electromagnetic simulation, the ZOR is verifiable as the metamaterial characteristics.

**Figure 4:**
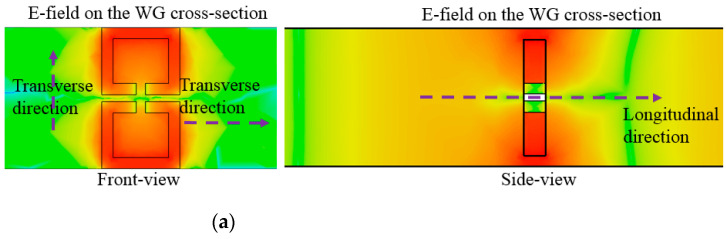

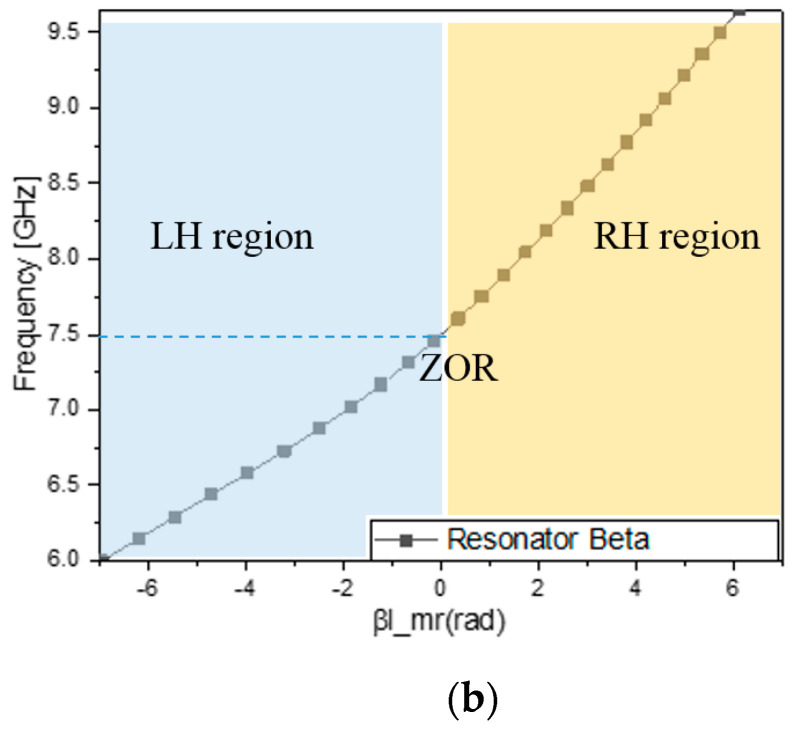
Observing the ZOR of the resonator: (**a**) E-field resonant and confined in a thin plane (**b**) dispersion diagram.

In the area of the conventional passive components, they resonate at the half-wave-long TX-line segment, and when becoming much shorter than the half wavelength, they are not resonant but evanescent. The field is strongly resonant on the structure that is far shorter than the half wavelength, as in Figure 4a. This field is observed at the target frequency. Especially in the side view, there are two vertical bars whose gap is roughly the half wavelength, and the thickness of the proposed resonator is a small fraction of a quarter wavelength. The electromagnetic wave entering the leftmost side (input port) propagates in the longitudinal direction, which is not blocked by this thin structure. Not as the evanescent mode, this propagation mode is consistent with the beta equal to zero occurring at the same frequency in reference of the dispersion diagram. Like the relationship between the beta (propagation constant) and the frequency, the nonlinear curve is formed from the LH (left-hand) region of the negative beta through the ZOR point to the RH (right-hand) region of the positive beta. For the conventional WG bandpass filters, cavities are designed as the half-wave-long waveguide sections for the resonance of interest and cascaded through irises for inter-resonator coupling. Generally, irises as H-plane or E-plane steps are not resonant but rather have reactance. On the contrary to that, the proposed resonator as the subwavelength structure passes the RF signal at the frequency. To generate a certain bandwidth as the passband and roll-off outside the band edges, resonators must be coupled for controlling the number of electromagnetic fields transmitted to the next resonator. This mechanism increases the order of the filter and the steepness of the skirt.

### 2.3. Formation of the Passband by Cascading the ZORs with Coupling Elements

The resonators are placed in series through coupling elements, as quantified in Figure 2 and Table 2. The coupling elements are denoted as *length_i_* for the eighth order of filtering. Prior to the high-order filter, the second-order BPF is built to see the basic characteristics of the coupling element suggested together with the ZORs.

**Figure 5:**
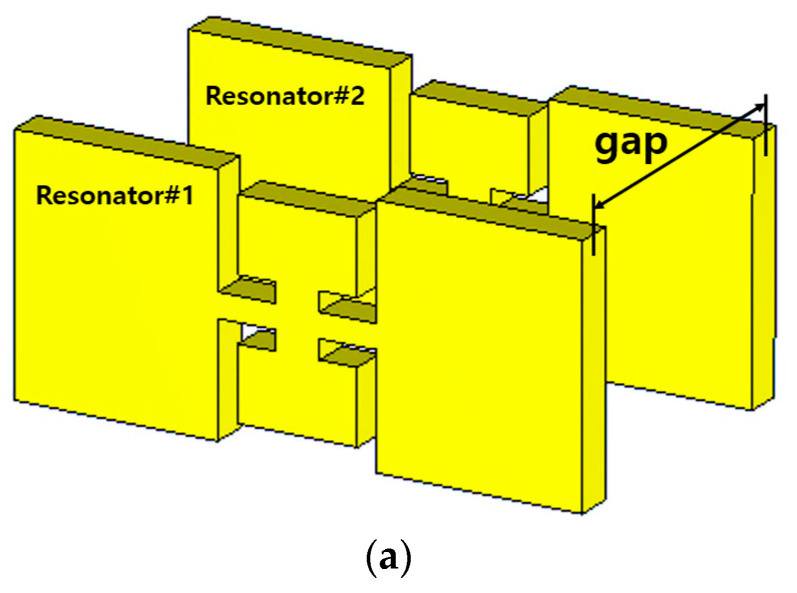

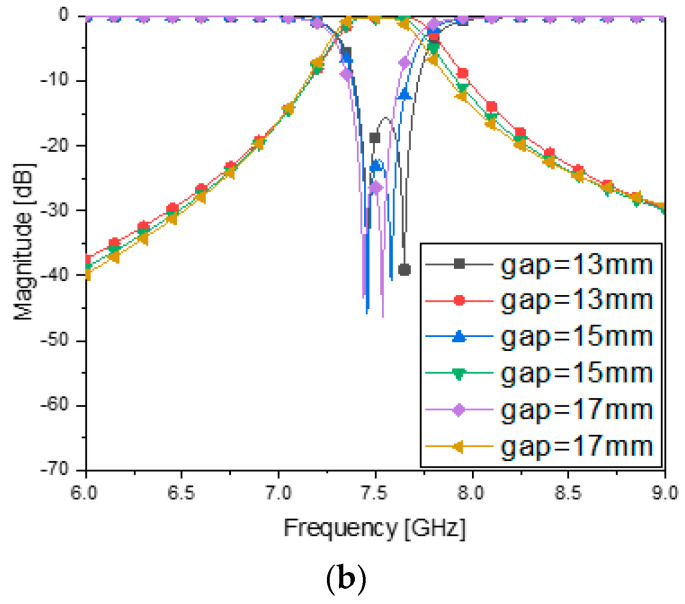
Combining two ZORs through the gap for coupling: (**a**) structure (**b**) S_11_ and S_21_ vs. gap.

Figure 5a is drawn with the geometrical parameters in Table 5. S_21_ in Figure 5b reveals that the slope of the skirt has been hastened compared to the one-pole case in Figure 3b. The gap length of the coupling element between the resonators is obtained by finding the value that generates the desirable S_11_ and S_21_ performances because it is varied, as in Figure 5b. The gap length of 15 mm is proper for the second-order case because the impedance matching S_11_ becomes worse; the bandwidth increases for a gap length of 13 mm, and the bandwidth decreases for gap length 17 mm. Now the attenuation level in the stopband is around 10 dB. To have higher attenuation at the stopband, the structure is extended to the eighth-order filter.

**Figure 6:**
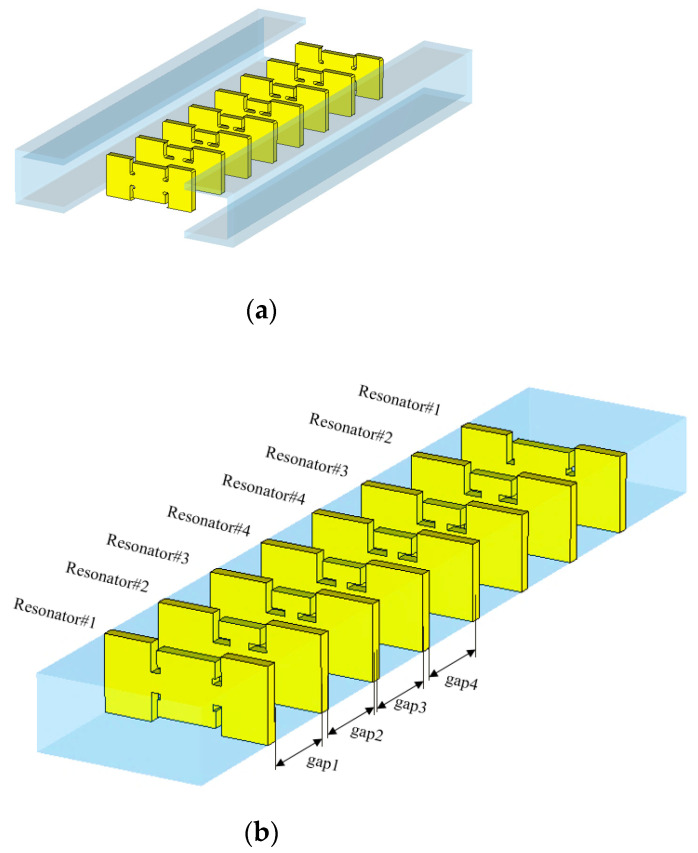

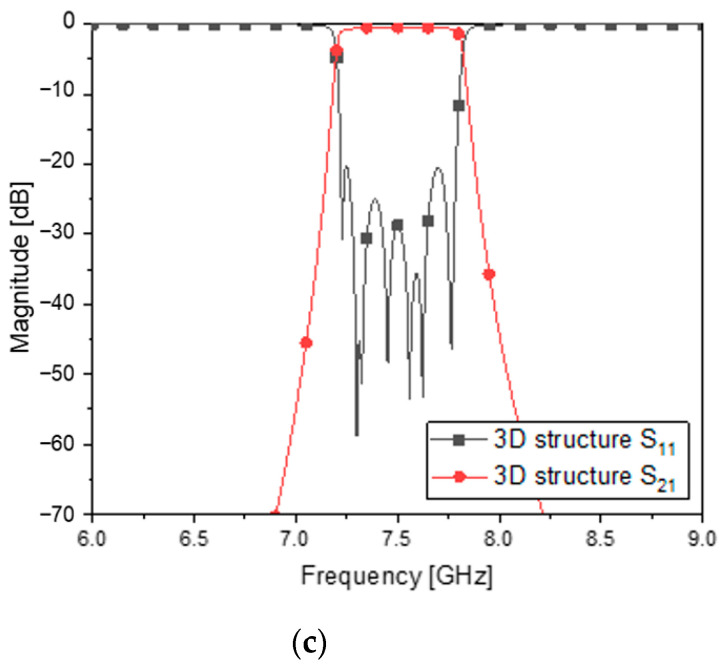
Combining eight ZORs through the gaps for coupling (**a**) open structure, (**b**) complete structure and (**c**) S_11_ and S_21_.

Beyond the two-pole BPF, for assuring a high level of noise suppression at the stopband, eight ZORs are put in order, and adjacent resonators are coupled through *length_ij_*. Figure 6a is an open structure before assembly, and Figure 6b is the complete shape. Giving the values to the variables as in Table 6, the full-wave EM simulation provides the designer with the frequency response of Figure 6c. Excellent impedance matching is peeked through an S_11_ of −20 dB and the insertion loss of an S_21_ of −0.9 dB in the passband. Among other things, the attenuation has been improved by a large margin with the steeper skirt. It is 40 dB.

## 3. Fabrication of the WG ZOR BPF and Testing the Prototype

The designed waveguide filter is fabricated and measured to validate the proposed method and geometry. Because the CNC-milling technique is conducted for fabrication, taking into account that the end-mill tip cannot realize the aforementioned shapes of the cross sections of the thin resonators 100%, the round corners appear instead of the sharp right-angle ones. Though the design has been performed with the unit of 10^-1^ mm as a coarse approach to ease the mechanical tolerance, the round corners are inevitable. The secondary procedure of design is performed to keep the function of the WG BPF satisfactory, as in Figure 6. This leads to the modified values for the geometrical parameters.

**Figure 7:**
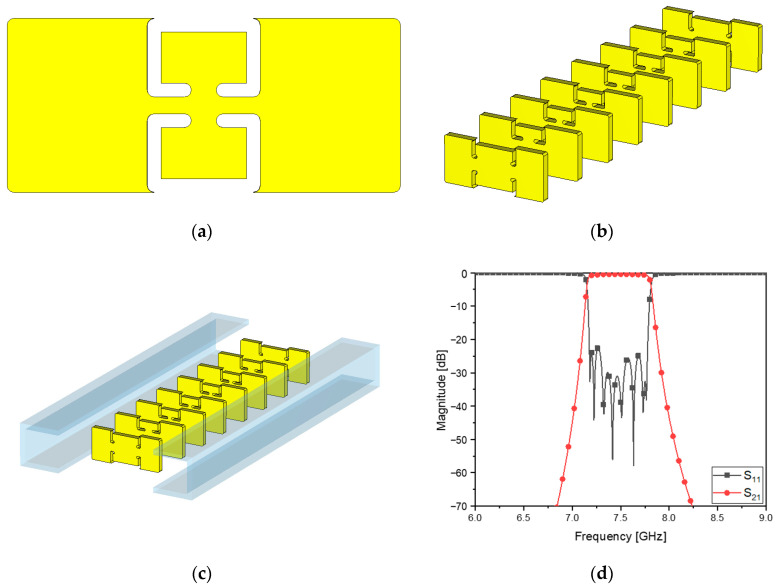
Realistic geometry of the eighth-order WG ZOR BPF: (**a**) front view of the thin resonator with the round corners, (**b**,**c**) 3D structure and (**d**) S_11_ and S_21_.

**Figure 8:**
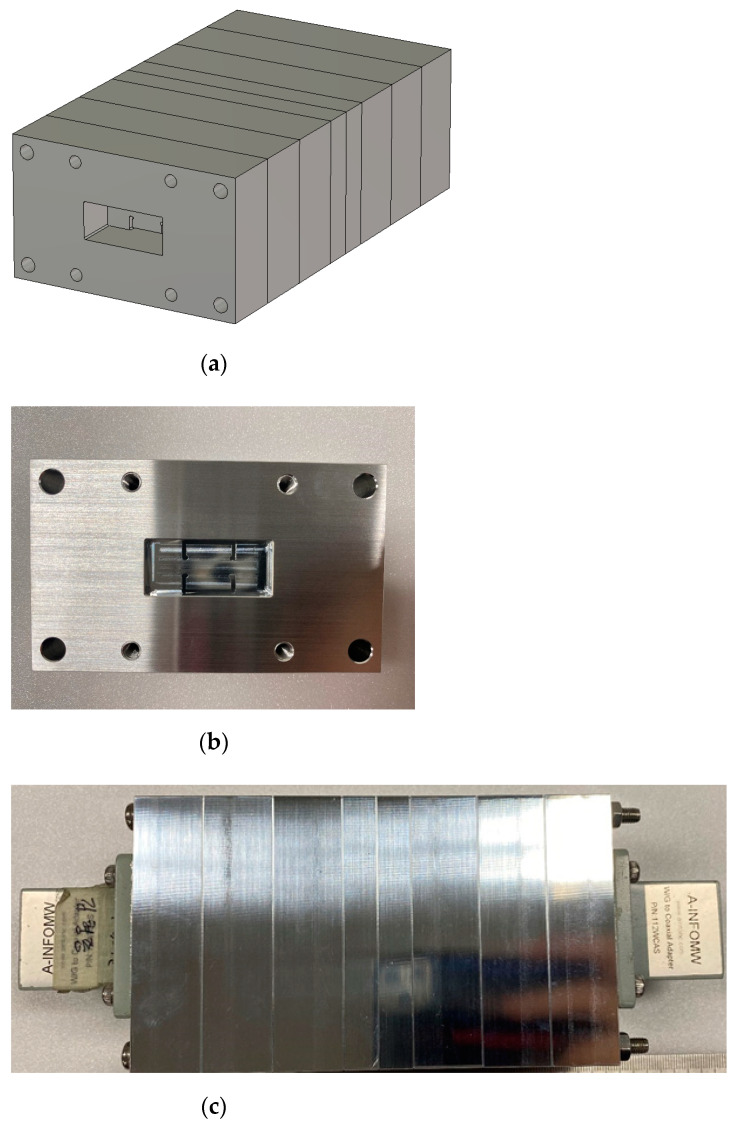

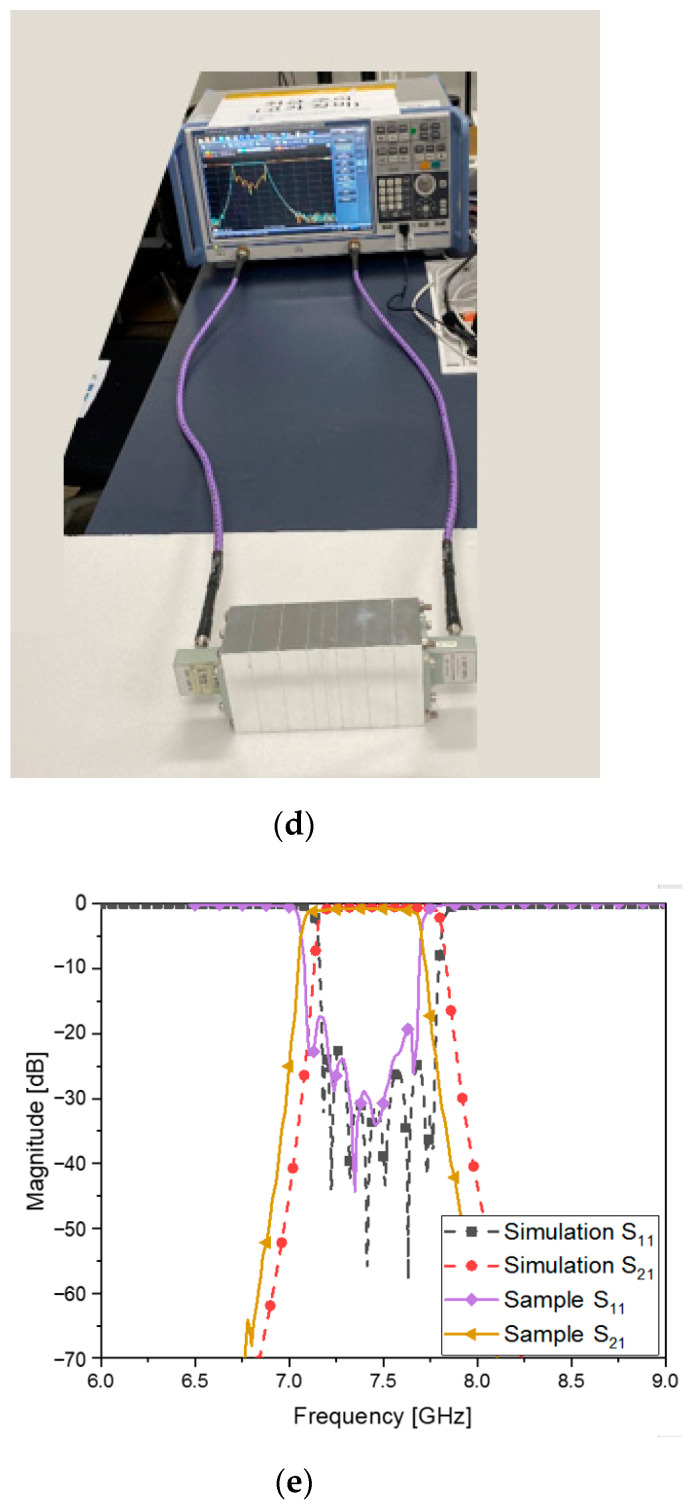
Prototyped eighth-order WG ZOR BPF via the CNC-milling process: (**a**) bird’s-eye view of the thin resonator with the round corners embedded in the flange body, (**b**) front view of the manufactured filter, (**c**) top view of the manufactured filter, (**d**) the device under test where the adaptors match with the flanges and (**e**) S_11_ and S_21_.

Despite the geometrical change in the front view of the flat metamaterial resonators, it is necessary to keep the frequency response compliant with the specifications. The corners become round and weaken the slot capacitance and the inductance on the edges of the metal patches and their intermediate strip. This means that at a microwave frequency band, it degrades the initial performance of the BPF. In the realistic case, Figure 3c and Figure 6 are rendered as in Figure 7a–c. Some of the physical dimensions have to be fine to be in the unit of 10^−2^ mm and no courser in order to achieve the required frequency response. Setting up the structure in the electromagnetic analysis software with the values for the geometrical parameters, the transmission and reflection coefficients are obtained as in Figure 7d, which meets the design requirement. This is very different from cavity filters presented by [15,16,17] in terms of shape and length, and it is physically realized as follows:

Following Table 7, the aluminum ingot is carved to the WG metamaterial resonators and coupling sections in the milling process as in Figure 8a, and they are pieced together to the eight-pole BPF as in Figure 8b,c. The original structure becomes a little longer and wider because of its having parts for mechanical assembly with holes, bolts and a flange body for WG-port connecting. However, the size of the core has the length of 14 cm for the eighth-order filtering. With this harness, the WG BPF is tested to determine the frequency response. This experiment conducted as in Figure 8d produces S_11_ and S_21_ as in Figure 8e. The insertion loss and the reflection coefficients are about −0.9 dB and −19 dB in the passband, respectively. The roll-off that this manufactured metamaterial filter makes is satisfactory with an attenuation of almost −40 dB. There occurs a discrepancy between the simulated and measured results such that the frequency is shifted downward a bit. It is inferred that connected pieces in the longitudinal direction do not tightly contact each other, making a tiny gap between metal rims with rotational misalignment. This proposed geometry goes through another novel approach for technical improvement. The remarkable size reduction enabled by the introduction to the waveguide metamaterial resonators meets the 3D-printing technique on the basis of fused deposition modeling (FDM), turning into extra weight reduction.

**Figure 9:**
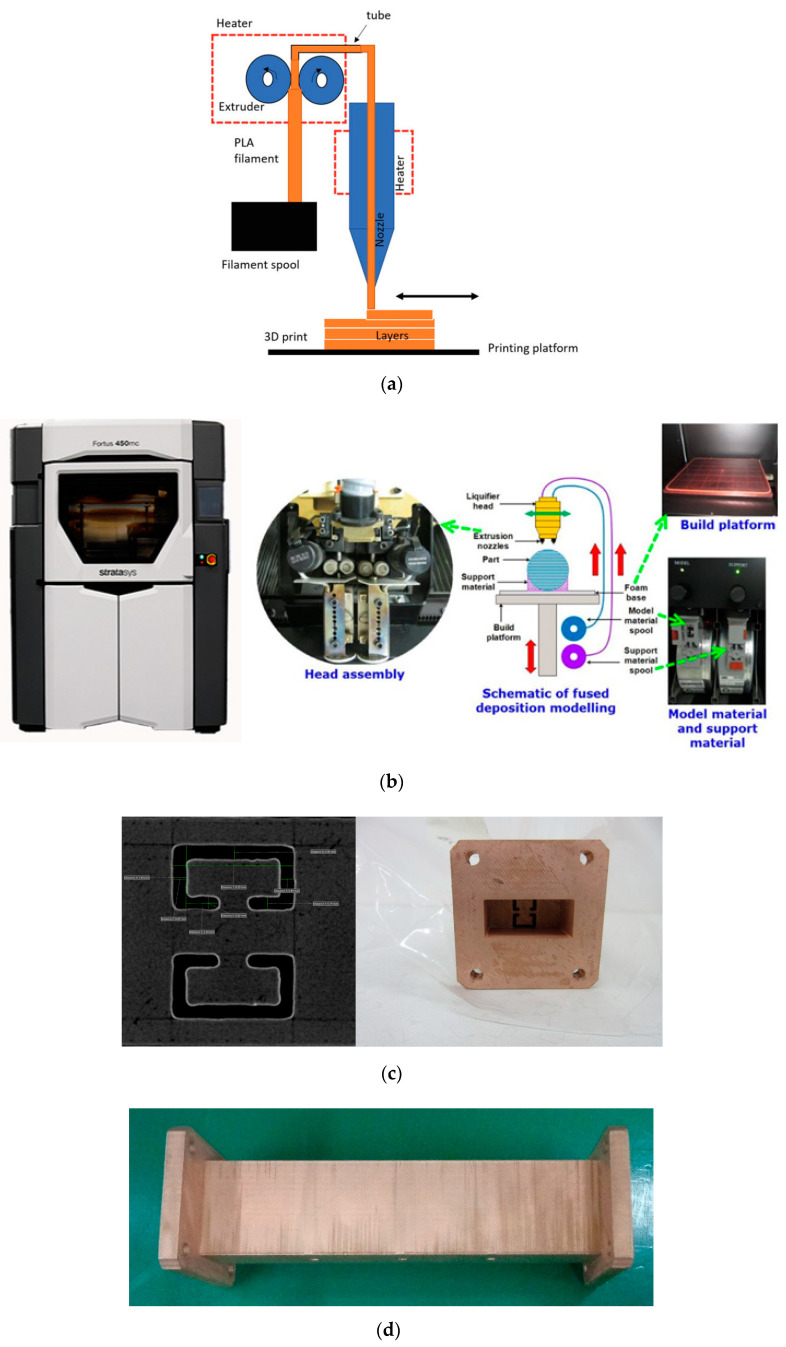

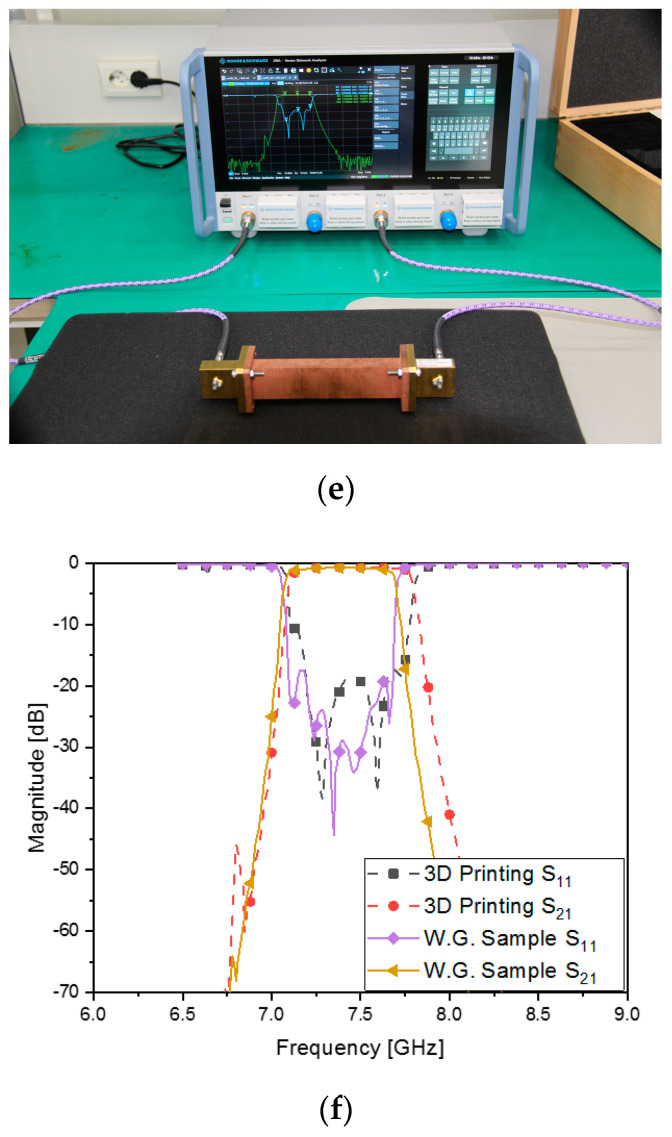
Prototyped eighth-order WG ZOR BPF via 3D-printing process: (**a**) basic idea of FDM [18], (**b**) the 3D printer employed here and its working mechanism [19], (**c**) front view of the inside garnished by the flange body, (**d**) top view of the 3D-printed filter, (**e**) our 3D-printed WG filter under test and (**f**) S_11_ and S_21_.

The metallic WG ZOR filter has been transformed to its 3D-printed version, manufactured as in Figure 9a, which was introduced by [18]. Using the 3D printer of FDM shown in Figure 9b, a polymer named PC (Polycarbonate) is additively grown to become the geometry appearing in Figure 9c,d owing to the filament projected by the tip of Stratasys (Fortus 450 mc) [19]. The inner and outer surfaces of the polymer are coated with copper, combined with electroless plating. PC is a superior plastic that has high strength, excellent fracture toughness and better heat resistance compared to conventional plastics, and it uses soluble supports for forming the structure. The produced 3D-printed structure is coated with the combined dry-and-wet fused metal coating process to have electrical properties. To improve the adhesion force, a plasma dry etching is applied to the pretreatment process. Then Pd (palladium) and Sn (tin) as reaction catalysts are used to improve the plating uniformity and adhesion strength of the electroless plating. Inside the rectangular body, the space consists of the ZOR resonators and coupling elements, as in Figure 8a,b. The novel structure is measured as in Figure 9e, whose s-parameters match those in Figure 9f, as the results: the electrical properties that are in good agreement with the CNC-milling-based WG BPF comply with the requirement. A small-scale error occurs, such as the upwards expansion of the upper band edge. It is assumed that the central resonators, such as numbers four and five, have the slots of the cross section disturbed by the surface roughness of the polymer and the discrepancies in width, length and thickness as blobs. Instead of one-round manufacturing, experiences in finding the right conditions for selecting the material, for positioning the tip, for the heat, for the density and for the time accumulated from repeated manufacturing will make the implementation better. Around 1362 g of the aluminum WG ZOR BPF has been lowered to approximately 54 g in the 3D-printed version. Both of the novel prototypes of the eighth-order bandpass filters are attractive to the placement in LEO satellites and microsatellites.

The characteristics of the proposed filter and reference BPF structures are compared as in Table 8. Most of all, the proposed filter has the shortest resonator as the WG metamaterial, which results in a good insertion loss from the complete structure at the length of 140 mm for a relatively low frequency, while [5,7] have lengths of around 128 mm and 231 mm each for a relatively high frequency. If the proposed method is applied to 11 GHz, the total length is expected to be 95 mm, which is shorter than [5] according to a quick estimation. This work, refs. [5,7] are high-order filters, giving high levels of attenuation in the stopbands, but [15,16,17] take four cavities, showing poor noise-suppression effects. If [15,16,17] are elongated to high-order filters, the lengths and insertion loss will be larger. Because the operation frequency of this work is much lower than others’ and has to use a WR-112 cross section as the largest, the 7.5-GHz filter might be the heaviest from Table 1 when the same order is assumed for all the compared cases. Thus, the total length must be as small as possible, enabled by the metamaterial resonators. The proposed filter is compared with the nonmetamaterial filter in terms of size and function.

**Figure 10:**
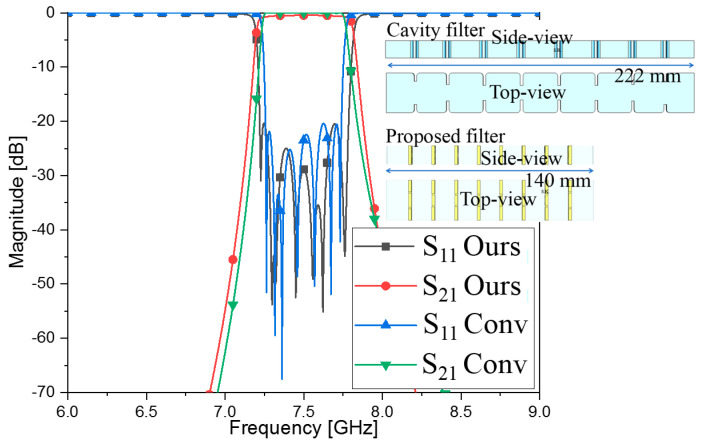
Comparing the proposed filter and nonmetamaterial filter with respect to geometries and frequency responses.

The cavity filter as a nonmetamaterial RF component is also designed to meet the requirement in Table 1. The total length of the conventional filter is 222 mm, and its frequency response is shown in Figure 10. S_11_ conv and S_21_ conv are satisfactory and are almost the same as the s-parameters of the proposed filter from the comparison. For the same cross section, the proposed filter is shorter than the conventional waveguide filter, as clearly seen in Figure 10.

## 4. Conclusions

A novel design method and geometry of the waveguide bandpass filter are suggested. Substantial size reduction and excellent bandpass filtering functions are made possible by coming up with the waveguide CRLH resonator, which leads to a very thin structure, much shorter than the half wavelength for the conventional cavities. The ZOR phenomenon is generated with the transverse geometrical parameters of the waveguide cross section, unlike other metamaterials, which utilize longitudinal line segments. The ZOR as the thin waveguide part does not block the incoming RF signal but instead passes it to the next ZOR. By cascading the ZORs through transmission sections as the coupling elements, the passband becomes distinct with the steeper skirt in the stopband. An eighth-order ZOR BPF is designed and simulated, moving to the stage of fabrication. It is manufactured into the aluminum waveguide filter and the PC 3D-printed WG ZOR BPF. The prototyped BPFs are measured and compared with each other and with simulated results. As to the passband, the insertion loss and the reflection coefficient are around ≤1 dB and ≤−15 dB, respectively, from simulation to measurement. The attenuation of ≥40 dB at 7 GHz and 8 GHz is achieved as desired in the specifications. The length of the WG ZOR BPF is 14 cm for the eighth pole, but the length of the conventional one is 20 cm even in the seven-pole case. To take a further step in decreasing the weight of the filter, the CNC-milling prototype and the 3D-printing technology are developed. The frequency responses are acceptable to use in the satellite transponder. The proposed filters make the LEO and scientific satellites much lighter, with a weight of 54 g, which is a weight that has been greatly reduced compared with the conventional WG filters.

## Figures and Tables

**Table 1:** Specifications the design will meet.

Item	Value
Insertion Loss	≤1 dB
Center Frequency	7.5 GHz
Bandwidth	500 MHz
Reflection coefficient	≤−15 dB
Out of Band Rejection (or Skirt)	≤−40 dB (fc ± 500 MHz)

**Table 2:** Calculated unknowns of Figure 1 and Figure 2 for the required BPF.

Variable	Value	Variable	Value
$L_{P1}$	0.998 nH	$L_{P2}$	0.516 nH
$L_{P3}$	0.417 nH	$L_{P4}$	0.408 nH
$C_{P1}$	0.442 pF	$C_{P2}$	0.859 pF
$C_{P3}$	1.064 pF	$C_{P4}$	1.088 pF
*length*1	20 mm	*length2*	15.6 mm
*length*3	16.6 mm	*length4*	16.5 mm
*length*5	16.6 mm		

**Table 3:** The values of the circuit elements of the E-CRLH resonator.

Variable	Value	Variable	Value
*L_R_*	1.6 nH	*C_R_*	2.27 pF
*L*1	0.586 nH	*C*1	0.626 pF
*L*2	1.51 nH		

**Table 4:** The values of the physical dimensions of the E-CRLH resonator.

Variable	Value [mm]	Variable	Value [mm]
*L*1	8.8	*W*1	1
*L*2	4.7	*W*2	1
*L*3	3.4	*W*3	1

**Table 5:** The values of the physical dimensions of the second-order filter.

Variable	Value [mm]	Variable	Value [mm]
*L*1	8	*W*2	1
*L*2	5.7	*W*3	1
*L*3	2.9	*gap*	15
*W*1	1		

**Table 6:** The values of the physical dimensions of the eighth-order filter.

**Resonator#1**			
**Variable**	**Value [mm]**	**Variable**	**Value [mm]**
*L*1	12.2	*W*1	1
*L*2	4.7	*W*2	1
*L*3	1.5	*W*3	1
**Resonator#2**			
**Variable**	**Value [mm]**	**Variable**	**Value [mm]**
*L*1	9.1	*W*1	1
*L*2	4.7	*W*2	1
*L*3	3.1	*W*3	1
**Resonator#3**			
**Variable**	**Value [mm]**	**Variable**	**Value [mm]**
*L*1	8.8	*W*1	1
*L*2	4.8	*W*2	1
*L*3	3.3	*W*3	1
**Resonator#4**			
**Variable**	**Value [mm]**	**Variable**	**Value [mm]**
*L*1	8.8	*W*1	1
*L*2	4.7	*W*2	1
*L*3	3.4	*W*3	1
**Gap**			
**Variable**	**Value [mm]**	**Variable**	**Value [mm]**
*gap*1	15.8	*gap*3	15.2
*gap*2	15.5	*gap*4	15.6

**Table 7:** The values of the physical dimensions of the eighth-order filter, factoring in fabrication.

**Resonator#1**			
**Variable**	**Value [mm]**	**Variable**	**Value [mm]**
*L*1	12.77	*W*1	1
*L*2	4.7	*W*2	1
*L*3	1.74	*W*3	1
**Resonator#2**			
**Variable**	**Value [mm]**	**Variable**	**Value [mm]**
*L*1	9.8	*W*1	1
*L*2	4.7	*W*2	1
*L*3	3.48	*W*3	1
**Resonator#3**			
**Variable**	**Value [mm]**	**Variable**	**Value [mm]**
*L*1	9.15	*W*1	1
*L*2	4.7	*W*2	1
*L*3	3.84	*W*3	1
**Resonator#4**			
**Variable**	**Value [mm]**	**Variable**	**Value [mm]**
*L*1	9.1	*W*1	1
*L*2	4.7	*W*2	1
*L*3	3.86	*W*3	1
**Gap**			
**Variable**	**Value [mm]**	**Variable**	**Value [mm]**
*gap*1	15.4	*gap*3	15.3
*gap*2	16.8	*gap*4	14.9

**Table 8:** Comparing the characteristics of the proposed filter and others’ filters.

	$f_{0}$ (GHz)	Insertion Loss (dB)	Attenuation (dB)	WG Flange	$\mathrm{Resonator}\;\mathrm{length}\;(\lambda_{g})$	Meta-Material
[5]	11	< 1	55	WR-90	0.68	X
[7]	11	< 1	50	WR-90	0.51	X
[15]	30	2	4.6	WR-28	0.57	X
[16]	9.45	0.08	>20	WR-90	0.51	X
[17]	8.175	0.35	7.9	WR-112	0.41	X
This work	7.5	0.9	40	WR-112	0.05	O

## Data Availability

Not applicable.
